# Granulomatosis with Polyangiitis Misdiagnosed as IgA Vasculitis in a Child

**DOI:** 10.1155/2023/9950855

**Published:** 2023-04-17

**Authors:** Sheida Amini, Mohsen Jari

**Affiliations:** ^1^Department of Pediatric, Imam Hussein Children's Hospital, Isfahan University of Medical Sciences, Isfahan, Iran; ^2^Department of Pediatric Rheumatology, Imam Hussein Children's Hospital, Isfahan University of Medical Sciences, Isfahan, Iran

## Abstract

**Background:**

Granulomatosis with polyangiitis (GPA) with early manifestations simulating IgA vasculitis is a very rare childhood systemic disease. *Case Presentation*. A 10-year-old boy presented initially with cutaneous, skeletal, and abdominal signs suggestive of IgA vasculitis. Over time, the worsening of skin ulcers, orchitis, and renal involvement led to the diagnosis of GPA according to cytoplasmic positive antineutrophil cytoplasmic antibodies and renal biopsy.

**Conclusion:**

Clinicians should be awared of the diagnostic pitfalls when making a clinical diagnosis of IgA vasculitis in children older than 7 years.

## 1. Introduction

IgA Vasculitis (Henoch–Schonlein purpura) is the most common childhood vasculitis. This vasculitis is characterized by the involvement of small vessels mostly in the skin, mesenteric vessels, and kidney tissue. IgA vasculitis usually presents with palpable purpura, arthritis, and abdominal pain in children aged 3 to 10 years. Although this disease usually has a self-limiting and benign course, in some rare cases, it may be associated with severe gastrointestinal manifestation, nephritis, and end stage renal disease [1]. Vasculitis associated with antineutrophil cytoplasmic antibody is rare in children. Increased disease severity, subglottic stenosis, and kidney disease are more commonly described in children [2]. What appears as IgA vasculitis initially may be a sign of a more serious vasculitis or vasculopathy, such as granulomatosis with polyangiitis (GPA, Wegener's Granulomatosis) or systemic lupus erythematosus. This case report was written to emphasize this issue so that misdiagnosis or delayed diagnosis in other cases can be avoided.

## 2. Case Presentation

**Figure 1:**
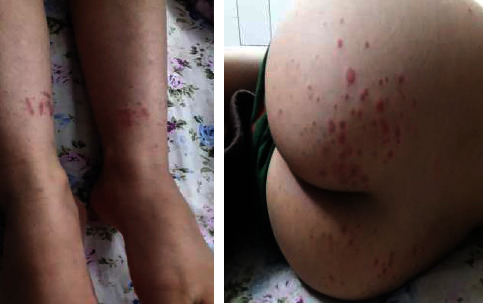
Palpable purpuric lesions on first admission.

**Figure 2:**
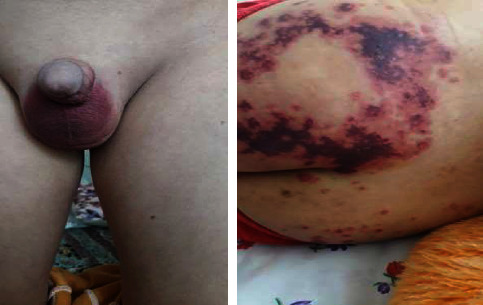
Three weeks later—orchitis and ulcerative lesions in the buttocks.

**Figure 3:**
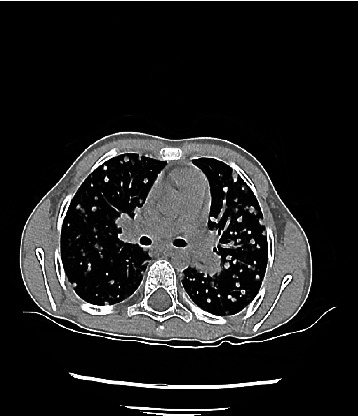
Multiple pulmonary nodules in both lungs on CT.

The patient was a 10-year-old boy who was admitted to the rheumatology department of Imam Hussein Children's Hospital, Isfahan University of Medical Sciences, Iran, due to abdominal pain and purpuric lesions in the buttocks and legs, swelling and pain in the knees, and abdominal pain in the preceding two days (Figure 1). On physical examination, palpable purpuric lesions in the mentioned areas and arthritis of both knees and general tenderness of the abdomen were found. The rest of the examination was normal. No previous hospitalization history was reported. The patient had no history of chronic nasal discharge, chronic sinusitis, or renal problems. Two years ago, the child had repeated epistaxis. He was discharged with a diagnosis of dry nose and a treatment with topical tetracycline ointment. Laboratory investigations showed a normal complete blood count (white blood counts: 11000/mm^3^, platelet: 380000/mm^3^, hemoglobin: 11.3 g/dL); erythrocyte sedimentation rate, C-reactive protein, urinalysis, and stool examination were within the normal range. Abdominal ultrasound and chest X-ray were normal. The patient was diagnosed as a case of IgA vasculitis. After two days of observation, due to the improvement of abdominal pain, he was discharged with the recommendation for follow-up and performed urinalysis weekly to visit the rheumatology clinic to measure blood pressure three weeks later. However, the patient returned to the hospital due to ulcerative lesions in the buttocks and orchitis (Figure 2). Laboratory tests were repeated. Complete blood count was normal, erythrocyte sedimentation rate = 34 mm/h, C-reactive protein negative, urinalysis showed blood 2+ and protein 2+, and 24-hour urine protein = 750 mg. Due to the flaring up of pre-existing disease and development of nephritis, the patient was further investigated for vasculitis. Perinuclear antineutrophil cytoplasmic antibodies (p-ANCA) were negative, antinuclear antibody was negative, blood complement was normal, and cytoplasmic ANCA (c-ANCA) = 185 U/mL (*N* < 12). According to the clinical and laboratory findings, the patient was diagnosed with vasculitis other than IgA vasculitis. A skin biopsy was performed for the patient. No evidence of immunoglobulin (IgA, IgM, and IgG) and complement deposition was seen. Leukocyte infiltration was present and the patient was diagnosed with leukocytoclastic vasculitis. As the history of frequent epistaxis and c-ANCA positivity was suggestive of GPA, chest computerized tomography scan was performed and showed multiple pulmonary nodules (Figure 3). The patient underwent a renal biopsy. Focal segmental glomerulosclerosis and features consistent with GPA were reported. According to the American College of Rheumatology criteria and the European League Against Rheumatism criteria, the diagnosis of GPA was confirmed. The patient was treated with cyclophosphamide pulse, methylprednisolone, and mycophenolate mofetil. On follow-up, three months later, the skin lesions had resolved. Urinalysis showed blood 1+, protein = 1+; 24-hour urine protein = 250 mg, and c-ANCA = 45 U/mL. Treatment was continued with monthly oral prednisolone, cyclophosphamide pulse, and mycophenolate mofetil (500 mg/m^2^). Six months later, physical examination was normal, c-ANCA < 12 U/mL, and urinalysis was normal. Treatment with oral prednisolone and mycophenolate mofetil was continued and the patient remained in complete remission.

## 3. Discussion

IgA vasculitis is the most common form of vasculitis in children. It causes inflammation and bleeding of the small blood vessels of the skin, kidneys, and other parts. The clinical signs and symptoms of IgA vasculitis may be indistinguishable from other forms of vasculitis [1].

GPA is a rare disease in that the blood vessels in multiple organs such as the testis, nose, sinuses, eyes, and kidneys become inflamed. There may also be a variety of cutaneous manifestations, the most common being purpura [3–5].

In the literature review, only 5 cases of GPA with an initial clinical diagnosis of IgA vasculitis were reported [6–10]. In those case reports, the primary manifestations were thought to be those of IgA vasculitis because of palpable purpuric lesions and renal involvement. But subsequent findings of positive c-ANCA and sinus and renal biopsy led to the correct diagnosis of GPA.

This case report is different from the previous cases. In addition to skin involvement, the patient had orchitis and severe ulceration in the buttocks area. The present case was also diagnosed within a short interval of three weeks, whereas in other reported cases, it took months if not years before the diagnosis of GPA was recognized.

## 4. Conclusion

Cautions should be taken when making a clinical diagnosis of IgA vasculitis in older children over seven years old with severe or atypical organ involvements as other rare vasculitides, including GPA, can mimic IgA vasculitis.

## Data Availability

No data were used to support the findings of this study.
